# USING BLACK HISTORY FOR BRAIN HEALTH: APPROACH, OUTCOMES, AND PUBLIC APPLICATIONS IN THE SHARP STUDY

**DOI:** 10.1093/geroni/igae098.1265

**Published:** 2024-12-31

**Authors:** Raina Croff

**Affiliations:** Chair

## Abstract

This symposium focuses on the Sharing History through Active Reminiscence and Photo-imagery (SHARP) study that centers Black history to engage older Black adults in the brain-healthy behaviors of walking, social engagement, and reminiscence within gentrifying areas of Portland and Seattle. Presentations span the study’s theoretical underpinnings to operationalizing results for public awareness of brain health. Dr. Raina Croff introduces the SHARP study, discussing the gap in brain health interventions that it seeks to fill, its culturally celebratory framework, and why such approaches matter for participant engagement. Anthony Cryer and Stephanie Johnson-Toliver discuss the collaborative process in Seattle from the community partner perspective, including recruitment challenges amidst gentrification, creating meaningful walking routes centered on local Black history, and participant feedback. Boeun Kim reports on health outcomes from the SHARP Seattle study and themes from evaluative focus groups, including barriers and motivators to engagement. Charles Fennell discusses the SHARP Caregiver Study in Portland, including adaptations to meet dementia family caregivers’ needs, and health outcomes around sleep and activity levels as captured by wearable digital biomarker technology. Taryn Gordon discusses preparing SHARP’s recorded walking narratives for a public digital oral history archive in a way that preserves participant integrity, and how SHARP concepts are operationalized in the SHARP website by integrating Black oral history from walking narratives with brain health information to educate and empower older Black adults to learn about and practice brain healthy behaviors.

